# A profile of volatile organic compounds in exhaled air as a potential non-invasive biomarker for liver cirrhosis

**DOI:** 10.1038/srep19903

**Published:** 2016-01-29

**Authors:** Kirsten E. Pijls, Agnieszka Smolinska, Daisy M. A. E. Jonkers, Jan W. Dallinga, Ad A. M. Masclee, Ger H. Koek, Frederik-Jan van Schooten

**Affiliations:** 1 Division Gastroenterology-Hepatology, Department of Internal Medicine, School for Nutrition and Metabolism Translational Research in Metabolism (NUTRIM), Maastricht University Medical Center, the Netherlands; 2 Department of Pharmacology and Toxicology, School for Nutrition and Translational Research in Metabolism (NUTRIM), Maastricht University Medical Center, the Netherlands; 3 Top Institute Food and Nutrition (TIFN), Wageningen, the Netherlands

## Abstract

Early diagnosis of liver cirrhosis may prevent progression and development of complications. Liver biopsy is the current standard, but is invasive and associated with morbidity. We aimed to identify exhaled volatiles within a heterogeneous group of chronic liver disease (CLD) patients that discriminates those with compensated cirrhosis (CIR) from those without cirrhosis, and compare this with serological markers. Breath samples were collected from 87 CLD and 34 CIR patients. Volatiles in exhaled air were measured by gas chromatography mass spectrometry. Discriminant Analysis was performed to identify the optimal panel of serological markers and VOCs for classifying our patients using a random training set of 27 CIR and 27 CLD patients. Two randomly selected independent internal validation sets and permutation test were used to validate the model. 5 serological markers were found to distinguish CIR and CLD patients with a sensitivity of 0.71 and specificity of 0.84. A set of 11 volatiles discriminated CIR from CLD patients with sensitivity of 0.83 and specificity of 0.87. Combining both did not further improve accuracy. A specific exhaled volatile profile can predict the presence of compensated cirrhosis among CLD patients with a higher accuracy than serological markers and can aid in reducing liver biopsies.

Cirrhosis is an advanced stage of liver fibrosis accompanied by vascular remodeling. It is the end-stage of chronic liver diseases, predominantly related to chronic viral infection, alcohol consumption, autoimmune and metabolic etiologies. Cirrhosis is usually characterized by an asymptomatic stage of compensated disease followed by a symptomatic stage of decompensated disease, defined by the presence of clinically evident complications^1^. These complications, *i.e.* ascites, variceal hemorrhage, hepatic encephalopathy, and jaundice, are associated with a markedly reduced life expectancy. Early diagnosis of compensated cirrhosis may prevent development of severe complications, *e.g.* by endoscopic screening of or prophylactic treatment for gastrointestinal varices^2^.

Although a combination of imaging together with impaired liver synthetic function is nowadays considered useful in clinical practice, liver biopsy is still the standard for a definite diagnosis^3^. Biopsy is, however, an invasive procedure associated with significant complications^4^, and not suitable for follow-up. Also, liver biopsies are prone to sampling error and inter-observer variation^5^,^6^.

Many studies evaluated non-invasive alternatives to grade liver fibrosis, including serum biomarker panels and radiological tests. Transient elastography (*i.e* FibroScan), for example, accurately detected cirrhosis in various chronic liver diseases with area under the receiver operating characteristic curve (AUC) values of 0.87–0.99^7^, but its local availability differs. The aspartate aminotransferase (AST) to platelet ratio (APRI) has a reported AUC of 0.83 with a sensitivity of 76% and a specificity of 72% in a meta-analysis of 40 studies including HCV patients^8^. FIB-4 is a serum biomarker panel that combines platelet count, alanine aminotransferase (ALT), AST and age and showed a high predictive accuracy for detecting cirrhosis (AUC 0.91) in HCV patients^9^. These promising serum biomarker panels use parameters available from routine laboratory tests and estimate disease severity, but do not necessarily reflect the dynamic changes, which are currently considered to be involved in fibrogenesis^7^, and/or hepatic metabolic function. Several breath tests using probe drugs as substrates have been applied to assess the functional metabolic capacity of the liver, but are easily affected by many factors, *e.g*. concomitant drug use and liver blood flow, especially in patients with chronic liver diseases^10^,^11^.

Analysis of volatile organic compounds (VOCs) in exhaled air was found to be useful for the diagnosis of several chronic diseases, including chronic inflammatory lung, cancer and intestinal diseases^12^,^13^,^14^,^15^,^16^. As the liver plays a key role in metabolizing endogenous and exogenous substances, liver damage may lead to accumulation of metabolites in the systemic circulation that can be excreted in breath^17^. Mainly pilot studies have identified exhaled VOCs to be associated with diseased liver^17^,^18^,^19^,^20^,^21^,^22^. Specific combinations of VOCs discriminated between patients with cirrhosis and healthy controls^20^,^21^. However, studies assessing the accuracy of exhaled VOCs in predicting the presence of cirrhosis within a heterogeneous group of chronic liver disease patients are still lacking.

Therefore, the primary aim of the present feasibility study was to identify an exhaled VOC profile for compensated liver cirrhosis that is able to discriminate chronic liver disease patients with from those without cirrhosis, independently from liver disease etiology and associated comorbidity, and to compare this with a panel of serological markers used in daily clinical practice.

## Methods

Consecutive patients with chronic liver diseases without (CLD, n = 87) or with compensated cirrhosis (CIR, n = 34) of different etiology and aged between 18–85 years were recruited from the Hepatology outpatient clinic between June 2010 and May 2012. Exclusion criteria were other known chronic gastrointestinal diseases [*e.g*. inflammatory bowel disease], chronic lung diseases [*e.g*. chronic obstructive lung disease, asthma, lung cancer)] and rheumatoid arthritis, or not willing to give informed consent.

Diagnosis of CLD and CIR was based on liver histology and/or clinical, laboratory, radiological and/or endoscopic findings. Compensated cirrhosis was defined by the absence of clinically evident complications (*i.e*. ascites, variceal hemorrhage, hepatic encephalopathy and/or jaundice). The severity of cirrhosis was assessed according to the Child-Pugh classification and Model for End-Stage Liver Disease (MELD) score. The liver diagnosis was performed before exhaled air analysis. Serological markers as part of clinical care were analyzed routinely by the hospital’s clinical chemistry laboratory and were retrieved from the computerized patient information system. The study population was a consecutive series of individuals defined by diagnostic and exclusion criteria.

Healthy volunteers (HC, n = 31) were recruited via local advertisement and considered eligible for inclusion when having a normal medical history, physical examination and liver tests [alaline transaminase (ALT) and γ-glutamyl transpeptidase (GGT)]. Further exclusion criteria were a history of gastrointestinal, liver and/or lung diseases, RA and a history of excessive alcohol consumption (*i.e*. >14 beverages per week).

The study was approved by the Medical Ethics Committee of Maastricht University Medical Center (MUMC), conducted according to the revised version of the Declaration of Helsinki (October 2008, Seoul) and registered at the US National Library of Medicine (http://www.clinicaltrials.gov, NTC01081236 and NTC01279356). All subjects provided written informed consent prior to participation.

### Sampling and analysis of exhaled air

Breath samples were collected by inflating a 5L Tedlar bag (SKC Ltd, Dorset, UK) and then transferred by use of a vacuum pump (VWR International, France) to carbon-filled stainless steel sorption tubes (Markes International, Llantrisant Business Park, UK) to trap the VOCs within 1 hour. Thermal desorption GC-*tof*-MS was used to measure the breath samples, as previously described^12^. The GC-*tof*-MS method applied here is a non-targeted GC-*tof*-MS method, i.e. before statistical analysis no prior identification of the compounds was performed.

### Data handling and statistical analyses

Subject characteristics are presented as median (range) and were compared between groups using the Mann-Whitney *U*-test for non-parametric data. Dichotomous variables were compared with the Chi^2^ test. A *P* < 0.05 was considered statistically significant using a two-tailed test. Before the actual statistical analysis the raw chromatograms obtained by GC-*tof*-MS (called breathograms) of HC, CLD and CIR patients were preprocessed to diminish various sources of artefacts before the actual statistical analyses, such as noise (i.e. rapid vertical fluctuations in the baseline), column bleeding, and chromatographic drift^23^.

**Figure 1:**
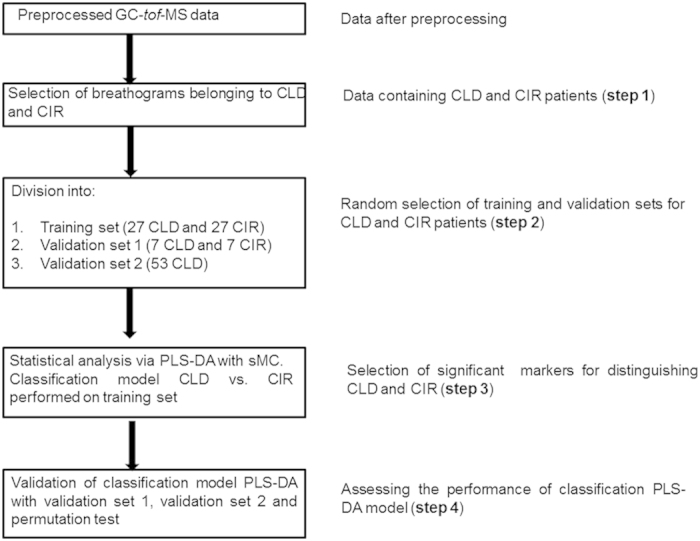
The conceptual flow chart of the statistical analysis. Steps 2 to 4 apply for serological markers and VOCs.

Routinely tested serological markers (Table 1) and VOCs in exhaled air were investigated for their ability to distinguish between CLD and CIR patients. We used well-established Partial Least Square Discriminant Analysis (PLS-DA) with Significant Multivariate Correlation (sMC)^24^ to identify serological markers or VOCs related to cirrhosis (Fig. 1, step 3)^23^,^25^. PLS-DA is a well-established multivariate tool commonly used in the field of metabolomics as well as in biomarker research^26^. PLS-DA constructs and optimizes a linear classification model based on the covariance between the serological markers or VOCs (the variables) and the final diagnosis (CIR or CLD), with optimal discrimination between the patient groups. The PLS-DA can be defined as a two equations model^27^, for data matrix **X** and corresponding vector of class membership **y**:


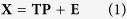



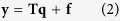


where, **T** is a score matrix containing orthogonal latent variables in the columns^24^, **E** and **f** are model residuals, **P** is **X** loading while **q** is **Y** loading. The relation between **X** and **y** can be expressed as:


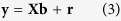


Where **b** is a vector of regression coefficient and **r** a vector of model residuals. The regression coefficient reflects the relative importance of the variables (here VOCs or serological markers) in predicting cirrhosis among CLD patients. Score matrix **T** will be used for the PLS-DA score plot. The combination of score matrix **T** and loading matrix **P** are used for PLS-DA bi-plot. The PLS-DA was applied on a training set of randomly selected 27 CIR and 27 CLD patients (Fig. 1; step 2). The prediction performance was established using a validation set containing 7 CLD and 7 CIR patients (randomly selected from the whole population, repeated 1000 times, i.e. validation set 1)^28^. For selecting training set and validation set 1, repeated stratified holdout (or stratified random subsampling) approach was used^29^. Finally, the significance of PLS-DA classification models was evaluated by the permutation test with 1000 iterations^28^.

The prediction accuracy of the PLS-DA classification models was graphically represented using the Receiver Operating Characteristic (ROC) curve, using group probabilities obtained from PLS-DA analysis. The probabilities for each sample add up by definition to 1 (*e.g.* 0.8 for CIR and 0.2 for CLD). The probabilities can be considered as a range of cut-off for which individual sensitivities and specificities are calculated and represented in the ROC curve. The optimal cut-off is used (here 0.505) to obtain the sensitivity and specificity of the PLS-DA model. The area under the curve (AUC) of the ROC curve is an indicator of predictive performance: a value close to 1 indicates high predictive power of the classification model. The prediction accuracy was also calculated for the remaining 53 CLD patients (*i.e*. validation set 2).

The outcome of the PLS-DA VOC classification model was visualized using a score plot and bi-plot. The score plot demonstrates the relations (*i.e*. similarity) between all patients. Each individual is is represented as a single point. Those points that lie close to each other have similar properties (i.e. here VOC profile), while points that are far away have different properties. In the bi-plot, information on the correlation between compounds and differences in relative abundance between CLD and CIR patients are presented.

## RESULTS

### Patients

Exhaled air was straightforwardly and safely obtained from all patients and HC. From the total of 121 patients, none was excluded from the analysis. No significant differences were observed for gender or smoking between both patients groups (Table 1). However, CIR patients were significantly older (*P* = 0.030), and had a lower BMI (*P* = 0.037) than CLD patients.

The etiology of the 34 CIR patients was alcohol (n = 9), chronic viral infection (n = 1), autoimmune-related (n = 7), metabolic (n = 4) and multifactorial or undefined in 13 patients. The Child-Pugh class of the cirrhotic patients was A in 24 and B in 10 patients, and the median international normalized ratio (INR) was 1.11 (0.95–1.51).

The etiology of the 87 CLD patients was metabolic (n = 28), autoimmune-related (n = 25), chronic active viral infection (n = 16), alcohol (n = 3) and multifactorial or undefined in 15 patients.

All 31 HC (52% male), with a median age of 47 (18–78) years and median BMI of 24.7 (18.1–32.1) kg/m^2^ had normal liver tests [GGT 18 (6–50) U/L and ALT 18 (10–32) U/L].

Drug therapy as standard medical care was given to all patients, including among others antibiotics (n = 3), glucocorticosteroids (n = 7), immunosuppressives (n = 9), laxatives (n = 11), proton pump inhibitors (PPI) (n = 43) and ursodeoxycholic acid (n = 53).

### Serological markers

**Figure 2:**
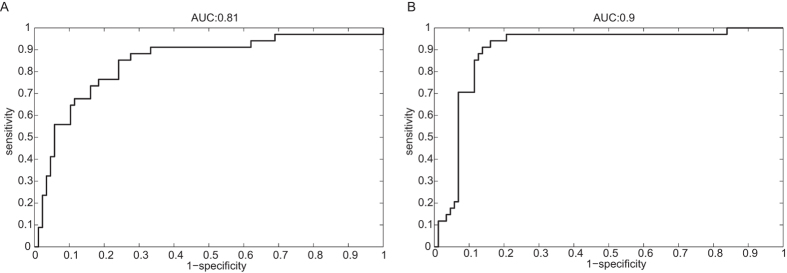
ROC curve for validation set 1 of the PLS-DA classification model obtained for CLD and CIR patients using; (**a**) 5 selected serological markers. AUC: 0.81 (95% confidence interval 0.77–0.91); (**b**) 11 discriminatory VOCs. AUC: 0.90 (95% confidence interval 0.86–0.96)

Liver functional mass was more impaired in CIR compared to CLD patients as shown by significantly lower ALT, GGT and albumin levels, and increased bilirubin levels (Table 1). The decrease of thrombocytes in cirrhotic patients may be the result of hypersplenism, which is indicative for the presence of portal hypertension. A combination of 5 markers, *i.e.* GGT, ALT, bilirubin, albumin, and thrombocytes, were the most discriminatory between CLD and CIR with a sensitivity and specificity of 0.71 and 0.84, respectively, and an AUC of 0.81 (95% confidence interval: 0.77–0.91) (Fig. 2a). Four of these serological markers were also statistically significant (Table 1). The permutation test using 1000 iterations led to a P-value of 0.001. The prediction of the 53 remaining CLD patients was 84.40%.

### VOCs

In total, 152 GC-*tof*-MS breathograms were measured (34 CIR, 87 CLD and 31 HC) and preprocessed^23^, resulting in a data matrix containing 3718 compounds (*i.e*. individual VOCs).

As proof of principle and to confirm previous publications^18^,^19^,^20^,^21^, we compared breathograms of HC with those of CLD and CIR patients. A set of 23 and 19 compounds enabled differentiating HC from CLD and CIR patients, with a correct classification of 100% and 93.75%, respectively (data not shown).

A VOC profile based on 11 compounds was identified using PLS-DA and allowed discriminating CIR from CLD patients. This PLS-DA model was then applied to the validation set 1, which contained; 7 CLD and 7 CIR. The corresponding ROC analysis showed sensitivity of 0.83, specificity of 0.87 and AUC of 0.90 (95% confidence intervals; 0.86–0.96) for the diagnosis of cirrhosis (Fig. 2b). The averaged negative (NPV), and positive predictive values (PPV) for CIR patients were 84.1% and 85.3%, respectively. The prediction of validation set 2, *i.e.* comprising the 53 remaining breathograms of CLD patients, was 86.8%.

**Figure 3:**
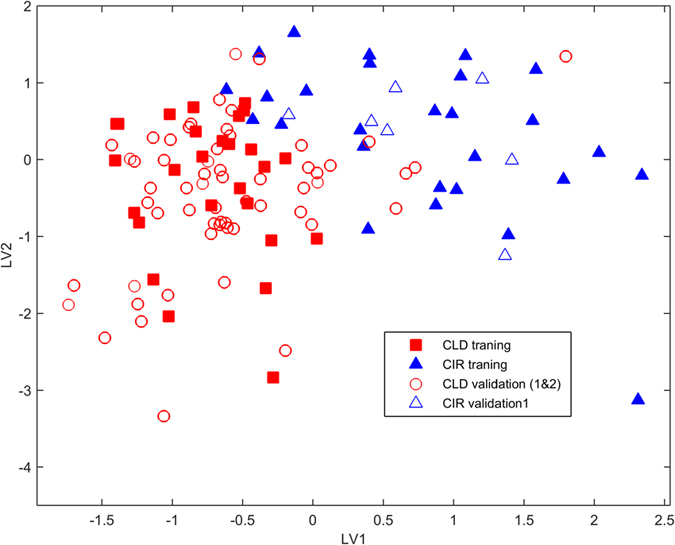
PLS-DA score plot of the final classification model obtained for CLD and CIR patients. Each patient is represented as a point (black filled square for training samples of CLD patients, grey filled triangle for training samples of CIR patients, black square for validation set 1 and validation set 2 of CLD patients, and grey triangle for validation set 1 samples of CIR patients). The two patient groups form distinct clusters indicating differences in VOCs profile.

The PLS-DA score plot for the VOCs is shown in Fig. 3. Here, two new variables (LV1 and LV2) were used to represent a linear combination of 11 discriminatory VOCs, showing the proportion of each VOC in discriminating between CLD and CIR patients. Each point represents a single patient, and color-coded for group membership (*i.e*. CLD and CIR) and marked as training or validation set. The samples of CLD and CIR patients of validation set 1 and 2 are projected accurately, *i.e.* within the cloud of the training samples belonging to the matching group. To further validate the obtained classification PLS-DA model, a permutation test was carried out using 1000 iterations and resulted in a *P*-value of 0.002.

Finally, to further test the significance of the set of 11 VOCs regularized Multivariate Analysis of Variance (rMANOVA)^30^ showed to be statistically significant (*P* < 0.001). The effect of etiology, drug therapy (*i.e*. antibiotics, glucocorticosteroids, immunosuppressive, laxatives, PPIs and ursodeoxycholic acid) and smoking in CLD and CIR patients on the selected set of discriminatory VOCs was also investigated using rMANOVA. No significant changes were found in selected VOCs with respect to etiology (P = 0.48), drug therapy (P = 0.23) and smoking (P = 0.12). The influence of alcohol could not be investigated, since only 3 patients were consuming alcoholic beverages at time of the study.

### Identification and relative abundance of VOCs

The 11 discriminatory VOCs were chemically identified by spectrum recognition using The National Institute of Standards and Technology library in combination with spectrum interpretation by an experienced mass-spectrometrist and identification based on retention times of compounds.

**Figure 4:**
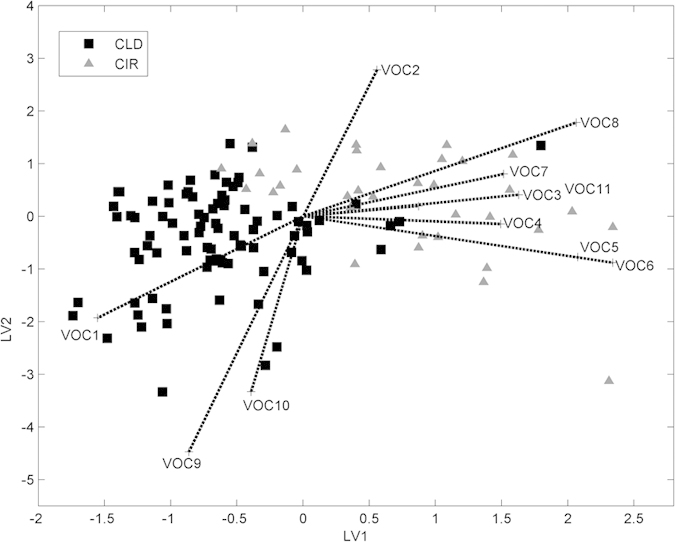
Bi-plot of PLS-DA analysis performed on 11 discriminatory VOCs in the breath of CLD (n = 87) and CIR patients (n =  34). Every point corresponds to a single breath sample and lines to the VOCs. VOC2 to VOC8 and VOC11 are found to be elevated in CIR patients. VOC1, VOC9, and VOC10 are reduced in CIR patients.

The PLS-DA bi-plot (Fig. 4) shows the change of compounds relative abundance between CLD and CIR patients. Each point corresponds to a patient, while the line to a compound. Correlation between compounds is reflected by the angle between the lines. For example, octane (VOC3) and propionic acid (VOC2) are positively correlated and their amounts are elevated in CIR compared to CLD patients. A negative correlation is found for propionic acid (VOC2) and 1-hexadecanol (VOC9) and the absence of correlation is found, for instance, between 3-carene (VOC6) and 1-hexadecanol (VOC9). The chemical identity of the discriminatory compounds and information about changes in relative abundances are listed in Table 2.

## Discussion

This study demonstrated that a combination of serological markers, *i.e*. GGT, ALT, bilirubin, albumin, and thrombocytes, can discriminate cirrhotic patients from patients with chronic liver diseases with a sensitivity of 0.71 and specificity of 0.84. A profile of 11 VOCs was found to predict the presence of cirrhosis with a sensitivity of 0.83 and a specificity of 0.87. The combination of both did not improve the diagnostic accuracy.

In our study we used GC-*tof*-MS to measure volatiles in exhaled air. This technique was combined with multivariate analysis based on established classification method PLS-DA. Next to GC-*tof*-MS, other analytical techniques can be used to measure various volatiles in exhaled air for instance Ion Mobility Spectrometry^31^, Proton Transfer Reaction – Mass Spectrometry^32^ or Selected Ion-Flow Tube Mass-Spectrometry^13^. All these analytical techniques have their advantages and disadvantages described elsewhere^33^,^34^. However, all can be combined with various machine learning techniques, such as PLS-DA, decision trees, Random Forests and Support Vector Machine, to identify most discriminatory patterns of compounds between cases and controls^35^,^36^. The choice depends on the characteristics of the data set. In the current study, optimal discrimination could be found by PLS-DA. However, independently form the applied statistical technique, the objective is always to unravel important information in the data.

To our knowledge, this is the first study that applied VOC analysis to predict the presence of compensated liver cirrhosis among CLD patients with different etiologies. We used an approach that analyzed the entire range of VOCs present in exhaled air. The classification model using 11 VOCs was very accurate in discriminating between CLD and CIR patients. Although, the numbers in the first validation set were small (7 versus 7, respectively), findings were further confirmed by correctly classifying 86.8% of the remaining 53 CLD patients in the validation set 2. Additionally, the permutation test was used to endorse the findings, indicating only 0.2% chance that the difference between groups was made by chance.

Our study confirmed that serological markers have a rather good diagnostic accuracy to detect liver cirrhosis^7^. Although, their specificity was comparable to VOCs, VOCs revealed a better sensitivity.

Half of the significant compounds appear to be hydrocarbons, including linear and branched alkanes, alkenes and aromatic compounds, which might be related to oxidative stress and/or an impaired metabolism by cytochrome P450 enzymes in the liver^20^. Furthermore, we found two different terpenes levels (C_10_H_16_ and terpenoid: α-pinene) increased in CIR compared to CLD patients, consistently with other studies^20^,^21^. An impaired liver metabolism could be involved as terpenes are metabolized by cytochrome P450 enzymes^20^. Similarly to previous studies, we found higher level of dimethyl disulfide in CIR patients, which may be generated by incomplete metabolism of methionine in the transamination pathway^20^,^37^.

In our population, cirrhosis was caused by alcohol in about 25% of patients and only 3 individuals still consumed alcohol at the moment of investigations. Thereby the direct toxic and metabolic effect of alcohol on the obtained results in this population is very unlikely. This is further supported by the lack of compounds identified related to alcohol consumption. A subgroup of patients was diagnosed with non-alcoholic fatty liver disease (NAFLD). Using a similar approach, Verdam *et al.*^22^, found n-tridecane, 3-methyl-butanonitrile, and 1-propanol to discriminate overweight subjects with from those without non-alcoholic steatohepatitis. These compounds were not discriminatory here, probably because of different populations included.

Not all discriminatory VOCs could be associated with pathological processes related to the liver. However, evidence is emerging that changes in the intestinal microbiota do occur in patients with liver diseases and their differential metabolic activity may be detected as volatile compounds.

In our study we tried to predict the presence of cirrhosis among a heterogeneous group of chronic liver disease patients, while in the majority of published studies patients with liver cirrhosis were compared to healthy controls. We also compared the VOCs from HC with the two patient groups, i.e. chronic liver disease with or without cirrhosis. Subsets of 23 and 19 compounds were able to differentiate CLD and CIR patients, respectively from HC with a correct classification of 100% and 93.75% in an independent validation set, thereby confirming previous studies^18^,^19^,^20^,^21^,^32^. The lack of overlap between these subsets and the 11 compounds discriminating between CLD and CIR indicates that in order to find sensitive and specific diagnostic markers for cirrhosis in patients with chronic liver diseases, profiles should be compared between CIR and CLD patients.

Liver biopsies were obtained in 70% of the patients. In the remaining patients cirrhosis was confirmed by evident clinical, laboratory, radiological and/or endoscopic findings, which is in line with current expert opinion of diagnosing cirrhosis^3^. Also, it has to be noted that some baseline characteristics differed between both patient groups. The higher age in CIR patients can be explained by disease progression and is inevitable when studying a representative sample from daily clinical practice. The lower BMI may be related to malnutrition and muscle wasting, often present in CIR patients^38^.

Another drawback is that not all discriminatory VOCs have full chemical identification. Although the chemical identification gives leads towards pathophysiological pathways, this was not the aim of the study and does require a different study design.

Furthermore, a large variety of VOCs are present in environmental air, but is not expected to be an important factor since all breath samples were taken randomly in time and at the same location.

The discriminating VOCs were also not affected by etiology, drug use and smoking as tested by rMANOVA. The effect of diet was not tested, as this information was not available for this cohort from daily clinical practice, and previous analysis could not find any relation between discriminatory VOCs and diet^39^,^40^. It should be noted that we deliberately included a heterogeneous group of patients to prove the concept that VOCs can be used to diagnose cirrhosis among patients with CLD independently of etiology, drug therapy and without standardization of dietary intake.

We investigated 121 patients with CLD. Other studies investigating liver cirrhosis with exhaled breath analysis usually included 60–70 individuals^21^,^22^,^32^,^41^. To strengthen our findings, we used an independent validation set as well as permutation test^28^. Although the sample size in this proof-of-concept study was reasonable, findings should be further validated using a large multicenter cohort with well-diagnosed patients. Moreover, it would be interesting to investigate the potential of VOCs to assess disease progression in prospective cohorts, as well as to study subgroups of patients with cirrhosis associated with complications or different stages of fibrosis, and to compare it for example with transient elastography.

VOCs can reflect liver metabolic function and, therefore, may provide leads towards pathophysiological pathways. Additionally, VOCs have a high short-term reproducibility^42^ and exhaled air collection is non-invasive, easy and requires only a minimal time investment. This technique is suitable to use in large cohorts, and could be implemented in clinical practice using sensor analysis. Much progress has been made in miniaturizing instruments that ultimately lead to manufacturing easy-to-use, cheap, hand-held equipment, in varying forms of sensors to detect volatile chemicals. Recently the electronic nose (e-Nose) has been shown to be of value in detecting acute liver failure in rats^43^. In humans, the e-Nose has already proven efficacy in other fields such as asthma and chronic obstructive lung disease^44^,^45^.

## Conclusions

Our findings indicate that the analysis of exhaled VOCs can more accurately predict the presence of compensated liver cirrhosis among CLD patients with different etiology than a panel of serological markers. Although the sensitivity and specificity should be further improved, the NPV of 84.1% indicates that VOCs may aid in reducing the number of liver biopsies in clinical practice.

## Additional Information

**How to cite this article**: Pijls, K. E. *et al.* A profile of volatile organic compounds in exhaled air as a potential non-invasive biomarker for liver cirrhosis. *Sci. Rep.* **6**, 19903; doi: 10.1038/srep19903 (2016).

## Figures and Tables

**Table 1:** Characteristics of patients

	CIR patients (n = 34)	CLD patients (n = 87)	*P*-value
Age (years)	59.5 (18–74)	54 (24–75)	**0.030**
Sex (M/F)	21/13	47/40	0.440
BMI (kg/m^2^)	25.3 (18.7–44.6)	28.3 (18.7–48.4)	**0.037**
Smokers	12	20	0.191
AF (U/L)	121 (46–230)	110 (58–680)	0.630
GGT (U/L)	54.5 (10–370)	69 (16–1337)	0.130
AST (U/L)	41 (13–135)	35 (11–487)	0.483
ALT (U/L)	28.5 (9–199)	46 (13–231)	**0.005**
Bilirubin (μmol/L)	18.9 (9–62.9)	13.4 (6.4–152.3) (n = 84)	**0.000**
Albumin (g/L)	37.4 (26.1–46.3)	40.2 (23.9–46.5) (n = 71)	**0.006**
Creatinine (μmol/L)	74 (40–108)	72.5 (13–113) (n = 80)	0.985
Thrombocytes x10^9^/L	112.5 (43–441)	233 (99–401) (n = 84)	**0.000**
Child-Pugh score*	5 (5–9)	—	—
MELD-score	8 (6–15)	—	—
Liver histology	24	47	—

Continuous values are presented as medians (range)

^*^One patient had a Child-Pugh score of 9, due to high bilirubin levels because of Gilbert’s Syndrome. None of the patients had clinically evident ascites, variceal hemorrhage, hepatic encephalopathy and/or jaundice.

**Table 2:** The identification of 11 discriminating VOCs.

Nr.	Chemical identity	Change
1	3-methylbutanal	(−)
2	Propanoic acid	(+)
3	Octane	(+)
4	Terpene (C_10_H_16_)	(+)
5	Terpenoid: α-pinene	(+)
6	3-carene	(+)
7	Unknown	(+)
8	Branched C_16_H_34_	(+)
9	1-hexadecanol	(−)
10	Branched C_16_H_34_	(−)
11	Dimethyl disulfide	(+)
